# Acute Appendicitis Following COVID-19 Infection in Pediatric Patients: A Single Center’s Study in Greece

**DOI:** 10.3390/diagnostics13122070

**Published:** 2023-06-15

**Authors:** Christos Kaselas, Maria Florou, Maria Tsopozidi, Ioannis Spyridakis

**Affiliations:** Second Department of Pediatric Surgery, Aristotle University of Thessaloniki, “Papageorgiou’’ General Hospital Thessaloniki, 54124 Thessaloniki, Greece

**Keywords:** acute appendicitis, COVID-19 infection, COVID-19 vaccination, pediatric surgery

## Abstract

Purpose: This study investigated the potential association between the previous severe acute respiratory syndrome coronavirus 2 (SARS-CoV-2) positive infection, as well as vaccination, and the presentation of acute appendicitis in pediatric patients. It has been three years since the World Health Organization (WHO) declared the SARS-CoV-2 pandemic, and city lockdowns and self-protective measures have been applied worldwide. In an effort to contribute to the research on the probable long-term complications of the COVID-19 infection as well as the vaccination against SARS-CoV-2, the current study was designed and investigated patients’ health records in the post-quarantine era. Methods: A retrospective analysis of patients admitted and treated surgically for acute appendicitis from January 2022 to June 2022 was conducted. Demographic and personal data, as well as the COVID-19 infection history of each child, were recorded. The patients who were negative for a previous COVID-19 infection were excluded. For the rest of the sample, the time-to-onset of acute appendicitis, the severity of appendicitis (complicated or uncomplicated), and the vaccination status of the patients were examined. Regarding the time-to-onset of appendicitis, we divided the patients into three groups: group A with a time-to-onset < 3 months, group B with a time-to-onset of 3–6 months, and group C with a time-to-onset of >6 months. Statistical analysis followed and was considered significant if *p* < 0.05. Results: Sixty-six children with a mean age of 10.5 years (range 1–15 years) were admitted for acute appendicitis during the determined period. After excluding 30 children that were negative for previous COVID-19 infection, we divided the patients into three groups: group A-23 children, group B-7 children, and group C-6 children. A statistically significant incidence of acute appendicitis diagnosis in <3 months after laboratory-confirmed COVID-19 infection (*p* < 0.01) was found. The incidence of complicated appendicitis was greater in patients with a positive SARS-CoV-2 history, with an estimated odds ratio of 1.8 (*p* > 0.05). The majority of the children (92%) had not received a COVID-19 vaccination. For the vaccinated children, the relative risk for complicated appendicitis was equal to 1.5 (*p* > 0.05). Conclusions: The results of our study demonstrate a potential positive relationship between COVID-19 infection and subsequent acute appendicitis in pediatric patients. There are also some speculations on the presentation of complicated cases of appendicitis following COVID-19 infection or vaccination, but these need to be further proven. Further data are required to better understand this potential complication of COVID-19 infection as well as the role of vaccines in the current post-vaccine era.

## 1. Introduction

Abdominal pain is a common complaint, and acute appendicitis is the most prevalent cause of acute abdomen among children that requires surgical treatment. The lifetime risk of acute appendicitis varies between 7% and 9%, with a peak incidence in the second decade of life [1]. According to the most prevalent theory, the luminal obstruction of the appendix induced by fecaliths, tumors, foreign bodies, and lymphoid hyperplasia leads to inflammation of the appendix. Lymphoid tissue is normally present in the appendix, behaves as a lymph node, and thus reacts to any viral infection or adjacent inflammation [2].

**Figure 1:**
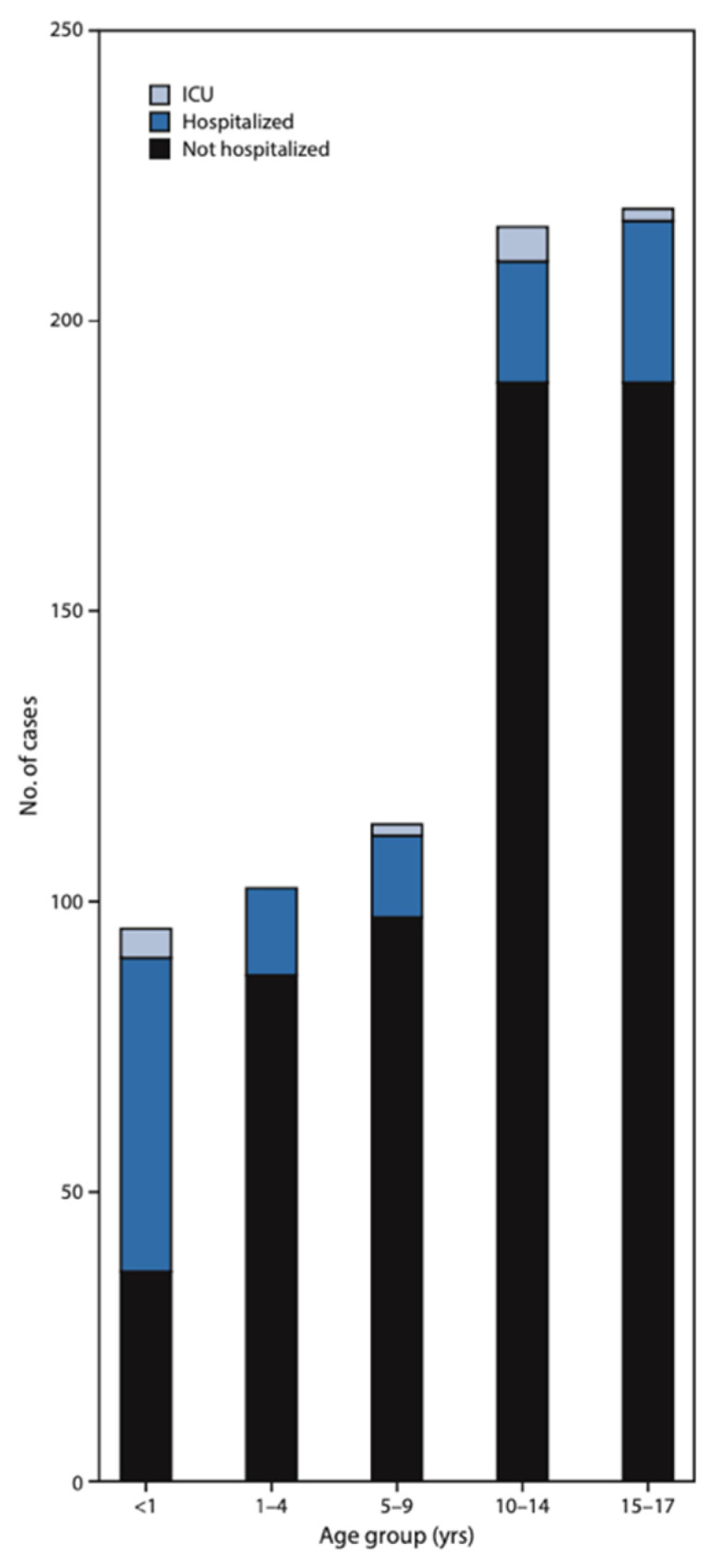
COVID-19 cases among children aged <18 years concerning their hospitalization status during the pandemic in the United States. Morbidity appears to be higher in young children aged < 4 years and especially in infants aged less than one year old [5]. ICU: intensive care unit.

Severe acute respiratory syndrome coronavirus 2 (SARS-CoV-2) presented at the end of 2019 in China and rapidly emerged as a great threat to the whole world. The World Health Organization (WHO) declared the SARS-CoV-2 pandemic in March 2020, along with strict stay-at-home orders, city lockdowns, and personal restrictive measures to prevent disease spread [3]. Children account for 1–8% of all confirmed cases of COVID-19 infection. They can be infected at any age, even in infancy, and usually have milder symptoms in comparison to adults [4]. Large epidemiologic studies from the United States and China reported that children of all ages are less likely to present with severe cough, fever, and dyspnea. They are also less likely to need hospitalization than adult patients, although admission rates and morbidity appear to be higher in young children aged <4 years and especially in infants aged less than one year old (Figure 1) [5,6,7]. Children with confirmed COVID-19 infection usually develop upper respiratory tract symptoms, such as cough, fever, headache, and runny nose, or the manifestations may be totally asymptomatic (Table 1) [5,6].

The gastrointestinal system can also be affected in approximately 25% of pediatric cases. The exact pathway of gastrointestinal manifestations induced by COVID-19 infection is still unclear. The detection of the SARS-CoV-2 RNA in the stool indicates the fecal-oral route of transmission. Furthermore, it has been stated that the angiotensin-converting enzyme type 2 (ACE2) receptor is a significant ligand of the S-protein on the surface of the SARS-CoV-2 virus particles and enhances its invasion into the hosts’ cells. The plentiful expression of ACE 2 receptors on the endothelial surface of the gastrointestinal system, especially in enterocytes, could serve as a secondary entry spot for the SARS-CoV-2 virus into the gastrointestinal tract [4,8]. Many symptoms, such as diarrhea, vomiting, abdominal pain, mesenteric adenitis, and appendicitis, have been recorded in SARS-CoV-2-positive patients, including adults and children [4,8]. Several studies have already reported a potential association between a COVID-19 infection and acute appendicitis, although there is also data supporting the exact opposite and not confirming any kind of relationship between these two clinical entities [9,10,11,12]. Patient behavior was also notably affected by the SARS-CoV-2 pandemic. It has been recorded that significantly fewer patients were asking for medical consultation from healthcare providers in order to avoid contracting the virus in hospital environments [5]. As a result, an increased incidence of complicated appendicitis was reported in most countries after the implementation of the city lockdowns and the stay-at-home advice [13]. There have also been some indications regarding the role of COVID-19 vaccines in the development of acute appendicitis as a hypothetical adverse event [14].

**Figure 2:**
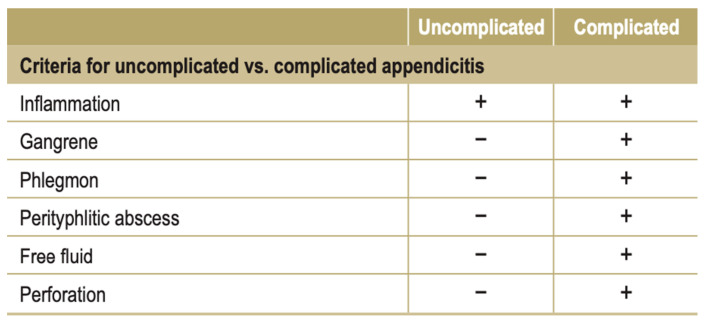
Overview of criteria for uncomplicated vs. complicated appendicitis, adapted from those of the European Association of Endoscopic Surgery [16].

Our study investigated the potential association between a previous COVID-19 infection and a subsequent acute appendicitis presentation in the current post-quarantine era, as the personal restrictive measures to control SARS-CoV-2 transmission are over and there are no stay-at-home orders anymore [15]. The acute appendicitis was classified as either a complicated case (inflammation of the appendix accompanied by periappendiceal phlegmon with or without perforation, gangrene, and a perityphlitic abscess) or an uncomplicated one (inflammation of the appendix in the absence of phlegmon, gangrene, free purulent fluid, or an abscess) (Figure 2) [16]. Furthermore, the role of the COVID-19 vaccines in the development of acute appendicitis was also investigated. Since last year, the COVID-19 vaccination among adolescents and children has been widely promoted in our country. More specifically, children and adolescents were immunized with mRNA vaccines of the type BNT162b2 introduced by Pfizer-BioNTech.

## 2. Materials and Methods

A retrospective analysis of all patients admitted and treated surgically for acute appendicitis during a period from January 2022 to June 2022 was conducted. The study took place in the academic pediatric surgery department of a tertiary teaching hospital. Demographic and personal data, as well as the SARS-CoV-2 history of each child, considering infection and vaccination, were recorded. The patients who were negative for a previous COVID-19 infection were excluded from the study. For the rest of the 36 patients, which is the actual study sample, the time-to-onset of acute appendicitis following COVID-19 infection, the severity of appendicitis (complicated or uncomplicated), and the vaccine immunization of the patients were examined. Regarding the mean time between a COVID-19 infection and the presentation of acute appendicitis symptoms, we divided the patients into three groups: group A with a time-to-onset < 3 months, group B with a time between 3–6 months, and group C with a time-to-onset of >6 months. The Excel software (Microsoft Excel, version 16.45, 2019 Microsoft) was used for data entry. The categorical variables were presented as numbers and percentages. All the continuous variables had a normal distribution and were presented as median values. The categorical variables were compared using the Chi-square test, applied in the Epi Info statistical program. The *p*-values < 0.05 were considered significant for the statistical analysis. The primary outcomes were the mean time between the COVID-19-positive infection and the acute appendicitis presentation, the severity of the appendicitis (complicated or uncomplicated), and the comparison between groups. The secondary outcomes were the vaccination history of the children and the possible relation to the incidence of acute appendicitis.

## 3. Results

In total, 66 children were admitted for acute appendicitis in our surgical department during the period from January 2022 to June 2022. The mean age of the sample was 10.5 years (range 1–15 years). We excluded 30 children who were negative for a previous COVID-19 infection or vaccination. Considering the time-to-onset of appendicitis following COVID-19 infection, the patients were divided into three groups: Group A—23 children that presented acute appendicitis in <3 months; Group B—7 children with acute appendicitis between 3–6 months; and Group C—6 children diagnosed with appendicitis in >6 months after COVID-19 infection (Table 2).

The comparison between groups found a statistically significant incidence of appendicitis in <3 months after COVID-19 infection (*p* < 0.01). There were some indications of a greater incidence of complicated appendicitis in patients with a positive COVID-19 infection history, but the estimated odds ratio was equal to 1.8 (*p* > 0.05) (Table 3). The majority of the children—92%—had not received a COVID-19 vaccination. For the rest of the vaccinated children, the estimated relative risk for complicated appendicitis was equal to 1.5 (*p* > 0.05) (Table 4).

## 4. Discussion

In the current retrospective analysis of patients during a six-month period that were admitted and treated surgically for acute appendicitis in our department, in relation to their personal history of COVID-19 infection, we estimated a significant incidence of appendicitis presentation in less than 3 months after COVID-19 infection (*p* < 0.01). Literature data on the association between a COVID-19 infection and acute appendicitis presentation are controversial. Similar results to ours were reported by Ljung et al. in 2022, as they estimated an increased risk of appendicitis after COVID-19 infection but not an overall association between COVID-19 infection and the specific time of onset of appendicitis [9]. On the other hand, there are studies that have failed to show any kind of relationship between COVID-19 infection and the time of onset of appendicitis in pediatric patients, such as this one by Jiang et al., who retrospectively investigated the personal histories of children treated for acute appendicitis, with emphasis on previous COVID-19 infection in the United States [11].

The primary finding of our study was the presentation of appendicitis less than 3 months after SARS-CoV-2 recovery. There are some possible explanations for this statistically significant finding. Lymphoid hyperplasia is a well-known immune reaction to viral infections anywhere in the body (e.g., tonsils, adenoids, the lymph nodes in the neck, and the lymph nodes in the abdomen). The lymphoid tissue of the appendix is more abundant in children and adolescents than adults and behaves as if it were in a lymph node. So, the lymphoid tissue of the appendix reacts to the SARS-CoV-2 viral infection, as in any kind of infection [2].

The induced hyperplasia of the appendiceal lymphoid follicle frequently causes luminal obstruction. Obstruction of the lumen is followed by swelling of the appendix, ischemia, rupture of the mucosal barrier, and, eventually, perforation and the presentation of acute appendicitis symptoms [17]. The viral-induced lymphoid hyperplasia and the subsequent appendicitis have been documented in the literature over the past decades and seem to be more common than generally accepted in the pathogeneses of the non-fecalith-induced appendicitis conditions [2,18].

On the other hand, temporary immunosuppression is an acknowledged condition following any viral infection [19]. It is acknowledged that the effectual T-cell reactions are critical to eliminating viral infections and preventing viral persistence. Functional early CD4 T-cell responses are important in triggering and maintaining CD8 T-cell activity through the production of effector cytokines such as interleukins (IL) and tumor necrosis factor-alpha (TNF–a) [19,20]. Over the years, viruses have generally developed many ways to escape the host’s immune system and consequently depress the host’s immune defense [20]. Viruses have learned to interfere with several mechanisms and cells of the immune system, such as T-lymphocytes, macrophage activity, interleukin-1 (IL-1), and T-cell growth factor (TCGF) production [19]. As far as SARS-CoV-2 is concerned, there are increasing clinical studies reporting that many alterations resulting in the functional impairment of the host’s cellular immune responses are also commonly observed in patients with COVID-19 infection [21,22]. Usually, patients with severe COVID-19 infections are found to have decreased immunological humoral reactions and compromised antiviral cellular immune responses due to the decreased number of lymphocytes, especially the subpopulations of CD4+ and CD8+ T lymphocytes [19,20]. Putting the pieces together, the COVID-19-induced depression of the immune system towards itself, as well as towards other unrelated pathogens, infections, and clinical conditions, could make the recovered patients vulnerable to a potential infection and inflammation of the appendix.

Contrary to the above-mentioned immune-suppression theory, an interesting explanation was given by Malhotra et al., who presented a novel association of acute appendicitis with previous COVID-19 infection in pediatric patients as a result of a hyperinflammation condition with gastrointestinal involvement. Similar to this approach, much attention has been drawn lately to the Multisystem Inflammatory Syndrome in Children (MIS-C) occurring a few weeks after COVID-19 infection, whether this is a completely asymptomatic COVID-19 infection or a severe pneumonia in children. MIS-C is a clinical entity characterized by a dysregulated immune system and hyperinflammation, resembling Kawasaki’s disease (KD), with a median age of presentation of 8.3 years. The cardiovascular system and the gastrointestinal system are the two most commonly affected systems, and the majority of pediatric patients with MIS-C require admission to the intensive care unit. Apart from the MIS-C, Malhotra et al. suggested acute appendicitis to be a separate late manifestation of COVID-19 infection as a post-infectious, hyper-inflammatory complication, with an occurrence approximately 2 weeks after infection [23].

Finally, there are some case reports recommending the systemic vascular manifestations of the SARS-CoV-2 disease as a probable cause of appendicitis. In theory, some uncommon adverse events of SARS-CoV-2 disease may be associated with small vessel inflammation and thrombosis. The virus-triggered injury of the endothelium of the vessels may be a probable explanation. So, lymphocytic phlebitis of the appendix as a microvascular complication of SARS-CoV-2 could possibly result in vascular occlusion of the appendix lumen and trigger the presentation of acute appendicitis symptoms [24].

Another primary finding of our study was the greater incidence of complicated acute appendicitis in patients with a positive SARS-CoV-2 history. Taking into account that our results were not statistically significant, this finding could indicate a potential long-term adverse event of COVID-19 infection by inhibiting the host’s immune system and leaving it susceptible to inflammation and infections [25,26]. Another possible explanation is the reluctance of patients to visit health care facilities out of fear of contacting the SARS-CoV-2 virus. Although our study took place in the post-quarantine period, when there were no restrictive stay-at-home orders, there is still significant reluctance among patients and parents to seek prompt medical advice because hospitals are still considered high-risk places for SARS-CoV-2 spread [25]. There is data supporting the fact that during the pandemic, the incidence of perforated acute appendicitis and peritonitis was remarkably increased, as were the length of hospital stays and the short-term and long-term postoperative complications, due to the delayed hospital visits [25,26]. Interestingly, Orthopoulos et al. reported in a 2021 study that during the pandemic, the incidence of complicated acute appendicitis increased while the overall incidence of acute appendicitis declined, indicating that maybe patients were not seeking prompt surgical consultation and management in that period [13]. On the other hand, there are studies that have failed to show any kind of pandemic influence on the development of appendicitis complications and morbidity in pediatric patients [27]. It should be underlined that Başkent et al. estimated significantly reduced negative appendectomy rates in the pandemic [27]. This finding is in accordance with the results by Orthopoulos et al., who mentioned a declining overall presentation of acute appendicitis patients in the emergency departments. Both studies point out that patients did not search for medical advice unnecessarily, unless it was a true emergency, during the pandemic [27]. In a large retrospective cohort study by Rodriguez et al., including only children and adolescents aged under 15 years old, the overall incidence and the disease progression of acute appendicitis during the quarantine period were examined. These data were then compared to hospital records of pediatric patients treated for acute appendicitis before the SARS-CoV-2 pandemic outbreak. They reported no significant differences regarding the incidence, the patients’ characteristics, the management (either surgical or conservative), the postoperative complications, or the related morbidity of children with acute appendicitis [28]. It is assumed that the differences among these aforementioned reports might be caused by the different restrictive measures that were applied in each country during the SARS-CoV-2 quarantine, the retrospective or prospective nature of the studies, the characteristics of the included patients (personal and family history, pediatric patients, adults in the sample, hospital policies for the management of patients), and the potential statistical bias.

Regarding the treatment of patients with acute appendicitis during pandemic periods, many healthcare facilities chose the conservative approach (administration of intravenous antibiotics and fluids, patients nil per os, and close monitoring) rather than the surgical one in stable patients with mild symptoms of acute appendicitis [29]. In our department, the management of appendicitis is surgical and has remained the same as before the pandemic outbreak: as soon as the diagnosis of acute appendicitis was established, an appendectomy was performed.

Concerning the secondary outcomes of our study, the majority of the children (92%) had not received a COVID-19 vaccination. In our country, children and adolescents were immunized with mRNA vaccines, specifically the type BNT162b2 introduced by Pfizer-BioNTech. The fact that the great majority of the sample was not vaccinated is no surprise, as COVID-19 vaccination hesitancy has been a common finding among parents in Europe, Asia, and the United States [30,31,32]. The level of appropriate parental information and understanding concerning the benefits and disadvantages of the vaccines is multifactorial and related to the socio-economic status, education, nationality, and religion of each family. The reasons associated with parental vaccination hesitancy, although they vary globally, mostly include concerns about the effectiveness, safety, and long-term complications of the vaccine, as well as the vaccine conspiracy theories that arose a little after the SARS-CoV-2 outbreak [30].

For the rest of the vaccinated children, the estimated relative risk for complicated acute appendicitis was elevated. Although our findings are not statistically significant, the review of the relevant literature revealed some variable data regarding the vaccines’ side effects and the appendicitis presentation. There are a few studies reporting that COVID-19 vaccination is associated with acute appendicitis onset, although it is rare, especially in younger populations aged 6–34 years old [33,34]. A large nationwide study from Israel investigated a variety of the vaccines’ adverse events, including pericarditis, arrhythmia, deep-vein thrombosis, pulmonary embolism, myocardial infarction, intracranial hemorrhage, thrombocytopenia, and appendicitis. The whole sample, consisting of the vaccinated group and the non-vaccinated control group, included 884,828 people. The researchers followed up on the patients for forty-two days after the application of the COVID-19 vaccine (day 0) and assessed a statistically significant risk ratio of acute appendicitis presentation following COVID-19 vaccination in the adult population [34]. Similar results were reported by Li et al. in a multinational network cohort study that evaluated the electronic health records of patients in eight countries in the United States, Europe, and Asia. They found the risk of appendicitis to be specifically increased in children and young adults [34]. On the other hand, a 2022 review by Pandit et al. reported no direct association between vaccines and appendicitis. They reviewed 111 studies consisting of pediatric populations aged 6–17 years and estimated the risk of a wide range of adverse events following COVID-19 vaccine administration. They described an incidence of appendicitis following COVID-19 vaccination that was not higher than expected within the general population [35]. Similar results were presented by Tankel et al., who studied the Danish population in 2021 and found no relationship between these two conditions [36].

## 5. Conclusions

To conclude, the preliminary findings of our study describe a potential positive association between COVID-19 infection and subsequent acute appendicitis in pediatric patients less than 3 months after SARS-CoV-2 recovery. There are also some indications in the presentation of complicated cases of appendicitis following COVID-19 infection and vaccination, but these indications cannot prove or disprove causality at the moment. Although our numbers are small and more studies are needed to verify this association, clinicians should be made aware of the possibility that acute appendicitis may present after SARS-CoV-2 recovery. Thus, a personal history positive for SARS-CoV-2 infection should be underlined by health care providers when examining a patient for an acute abdominal pain complaint. Further studies on pediatric populations will be needed to fully evaluate the risk of acute appendicitis following COVID-19 infection, as well as the safety and potential adverse events after a COVID-19 vaccination.

## Figures and Tables

**Table 1:** Signs and symptoms among children and adolescents (age < 18 years) with laboratory-confirmed SARS-CoV-2 disease [5].

Sign/Symptom	No. (%) of Pediatric Patients
Fever	56%
Cough	80%
Shortness of breath	39%
Myalgia	7%
Runny nose	21%
Sore throat	24%
Headache	28%
Nausea/Vomiting	11%
Abdominal pain	6%
Diarrhea	13%

**Table 2:** Patients with acute appendicitis and their COVID-19 infection history.

Patients with	COVID Infection History	No COVID Infection History
Appendicitis, *n* (%)	36 (100%)	30 (100%)
<6 months	30 (83%)	3 (10%)
3–6 months	7 (19%)	-
<3 months	23 (64%)	-
>6 months	6 (17%)	27 (90%)

**Table 3:** Severity of appendicitis regarding the COVID-19 infection history.

Patients with	COVID Infection (+), *n* (%)	COVID Infection (−), *n* (%)
Appendicitis, *n* (%)	36 (100%)	30 (100%)
Complicated	6 (7%)	27 (90%)
Uncomplicated	30 (83%)	3 (10%)

**Table 4:** Severity of appendicitis regarding the COVID-19 vaccination status.

Patients with	Vaccination	No Vaccination
Appendicitis, *n* (%)	5–8%	61–92%
Complicated	1	8
Uncomplicated	4	53

## Data Availability

Not applicable.
